# Supplementary material for: Application of Raney Al-Ni Alloy for Simple Hydrodehalogenation of Diclofenac and Other Halogenated Biocidal Contaminants in Alkaline Aqueous Solution under Ambient Conditions
Source: Materials (Basel). 2022 May 31;15(11):3939. doi: 10.3390/ma15113939 (PMC9182476; doi:10.3390/ma15113939)
Supplement: Supplementary file 1 [file materials-15-03939-s001.zip › materials-1708852-supplementary.pdf]

Supplementary Materials

Application of Raney Al-Ni alloy for simple hydrodehalogenation of Diclofenac and other halogenated biocidal contaminants in alkaline aqueous solution under ambient conditions

Helena Bendová[1], Barbora Kamenická[1], Tomáš Weidlich[1,*], Ludvík Beneš[2], Milan Vlček[2], Petr Lacina[3], Petr Švec[4]

[1] Chemical Technology Group, Institute of Environmental and Chemical Engineering, Faculty of Chemical Technology, University of Pardubice, Studentská 573, Pardubice CZ-532 10, Czech Republic
[2] Joint Laboratory of Solid State Chemistry, Faculty of Chemical Technology, University of Pardubice, Studentská 573, Pardubice CZ-532 10, Czech Republic
[3] GEOtest, a.s., Šmahova 1244/112, Brno CZ-627 00, Czech Republic
[4] Department of General and Inorganic Chemistry, Faculty of Chemical Technology, University of Pardubice, Studentská 573, Pardubice CZ-532 10, Czech Republic

Corresponding author: Tomáš Weidlich, E-mail: tomas.weidlich@upce.cz

Keywords: nickel alloy; reductive dechlorination; hydrometallurgy; $NaBH_4$; biocide; drug; water treatment

Experimental section

Preparation of APA:

The standard of 2-anilinophenylacetic acid (APA) was prepared by hydrodechlorination of DCF according to the experimental procedure described in the main document using a higher concentration of reactants: 100 mL of 25 mM NaDCF (2.5 mmol), 540 mg of Al-Ni (10 mmol Al) was added and obtained suspension was mixed with 100 mL of 250 mM aq. NaOH (50 mmol) and stirred for 240 minutes. Subsequently, the decanted aqueous phase was added to 100 mL of 16wt.% H_2SO_4 and 1M aqueous NaOH was added to the obtained acidic solution dropwise to precipitate APA. The precipitated APA was isolated by suction, washed with water and dried in air. The product was analyzed by ^1H and ^{13}C NMR spectroscopy.

NMR spectroscopy:

The solutions were obtained by dissolving approximately 20 mg of each compound in 0.6 ml of deuterated solvent. The values of ^1H chemical shifts were calibrated to residual signals of $CDCl_3$ ($\delta(^1H)$ = 7.27 ppm) or DMSO-d_6 ($\delta(^1H)$ = 2.50 ppm). The values of ^{13}C chemical shifts were referred to signals of DMSO-d_6 ($\delta(^{13}C)$ = 39.52 ppm) or $CDCl_3$ ($\delta(^{13}C)$ = 77.23 ppm). Positive chemical shift values denote shifts to the higher frequencies relative to the standards.

The attribution of resonances in both ^1H and $^{13}C\{^1H\}$ NMR spectra was carried out according to the literature data available for Diclofenac (DCF) and 2-anilinophenylacetates (APA) [54–57] or using SDBS NMR spectra database [58]. See Fig. S1 for the general NMR numbering of DCF and APA.

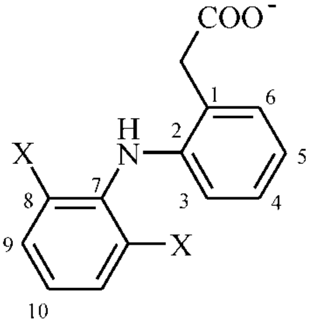

Figure S1. General NMR numbering of DCF (X = Cl) and APA (X = H).

Measured multinuclear NMR spectra of DCF and APA are depicted in Figure S2–S9. Both multiplicity and integral intensities of all resonances are in accordance with the proposed structures. Based on observed resonances of starting DCF and the corresponding product of its hydrodechlorination (HDC), it is evident that the HDC is quantitative, providing pure APA as the sole product (valid for Table 1 in the manuscript, run 4-10 and 12).

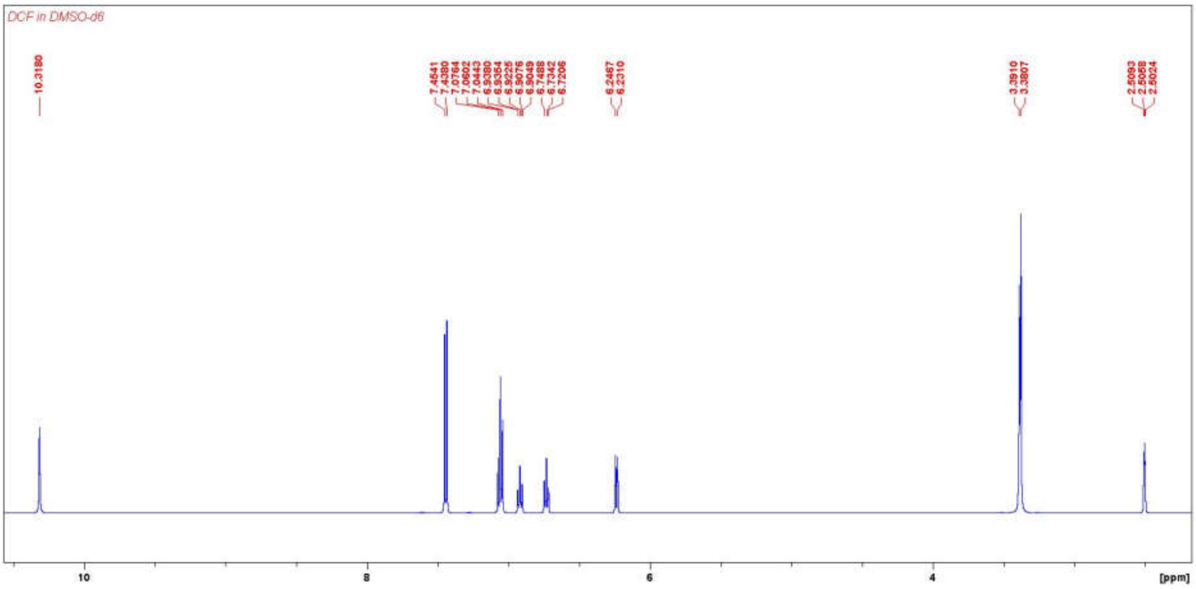

Figure S2. ^{1}H NMR spectrum of DCF in DMSO-d_6. δ (ppm): 10.32 (s, 1H), 7.44 (d, 2H), 7.06 (m, 2H), 6.91 (t, 1H), 6.73 (t, 1H), 6.24 (d, 1H), 3.39 (s, 2H). Resonances at 3.38 and 2.50 ppm correspond to water and residual DMSO, respectively.

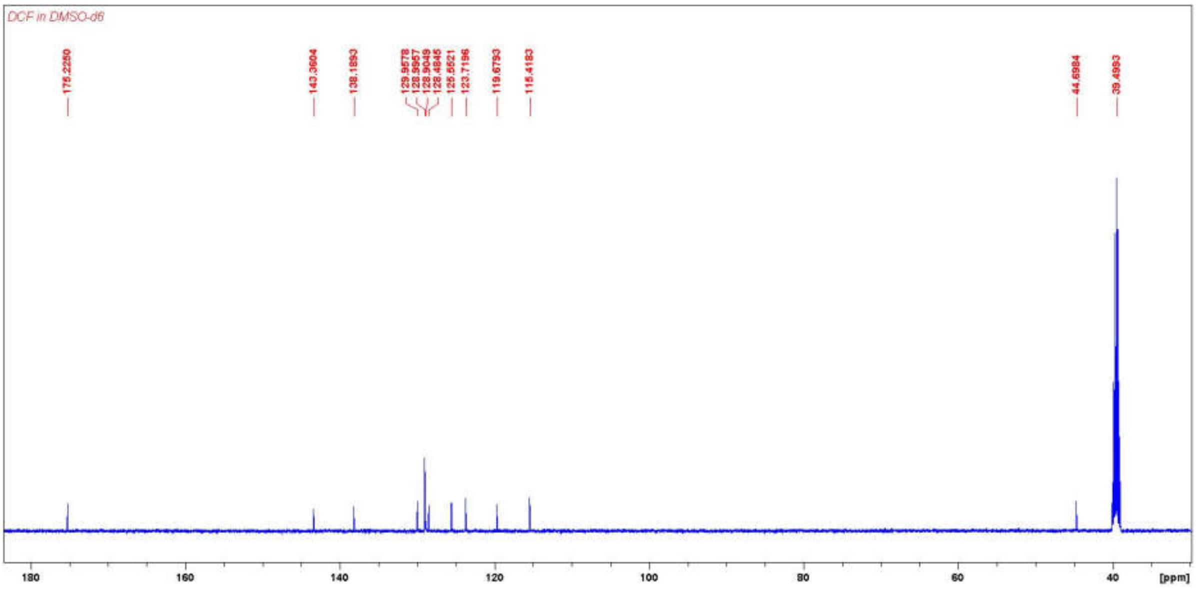

Figure S3. $^{13}C\{^{1}H\}$ NMR spectrum of DCF in DMSO-d_6. δ (ppm): 175.2, 143.4, 138.2, 129.9, 129.0, 128.9, 128.5, 125.6, 123.7, 119.7, 115.4, 44.7. Resonance at 39.5 ppm corresponds to DMSO.

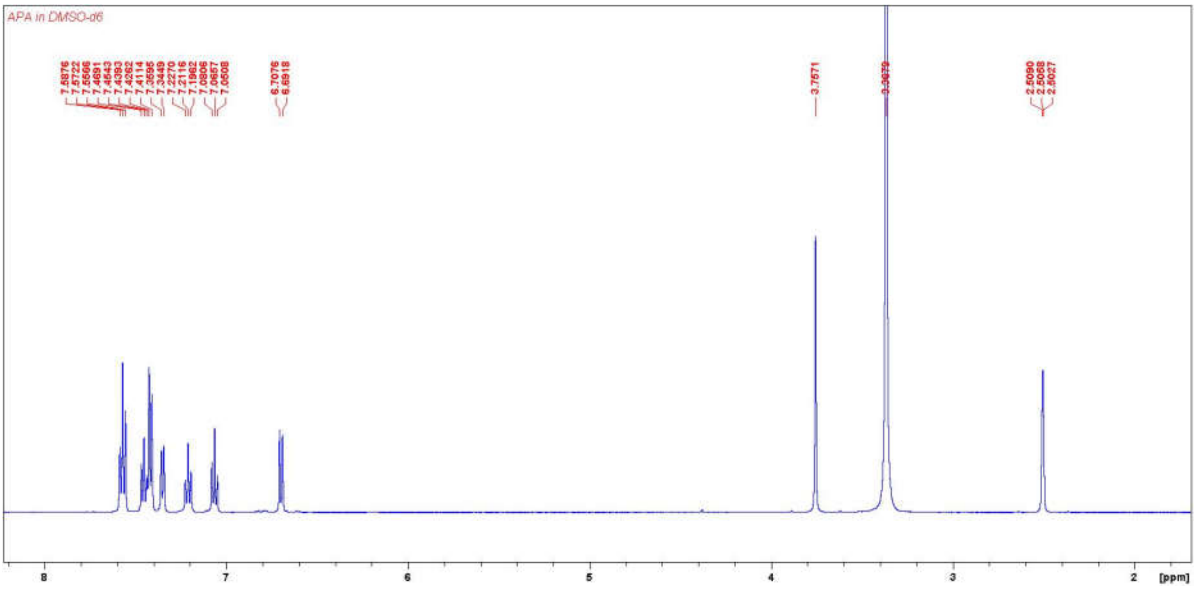

Figure S4. ^{1}H NMR spectrum of APA in DMSO-d_6. δ (ppm): 7.58 (m, 2H), 7.45 (t, 1H), 7.42 (d, 2H), 7.35 (d, 1H), 7.21 (t, 1H), 7.06 (t, 1H), 6.70 (d, 1H), 3.75 (s, 2H). Signal of the NH fragment was not observed. Resonances at 3.37 and 2.50 ppm correspond to water and residual DMSO, respectively.

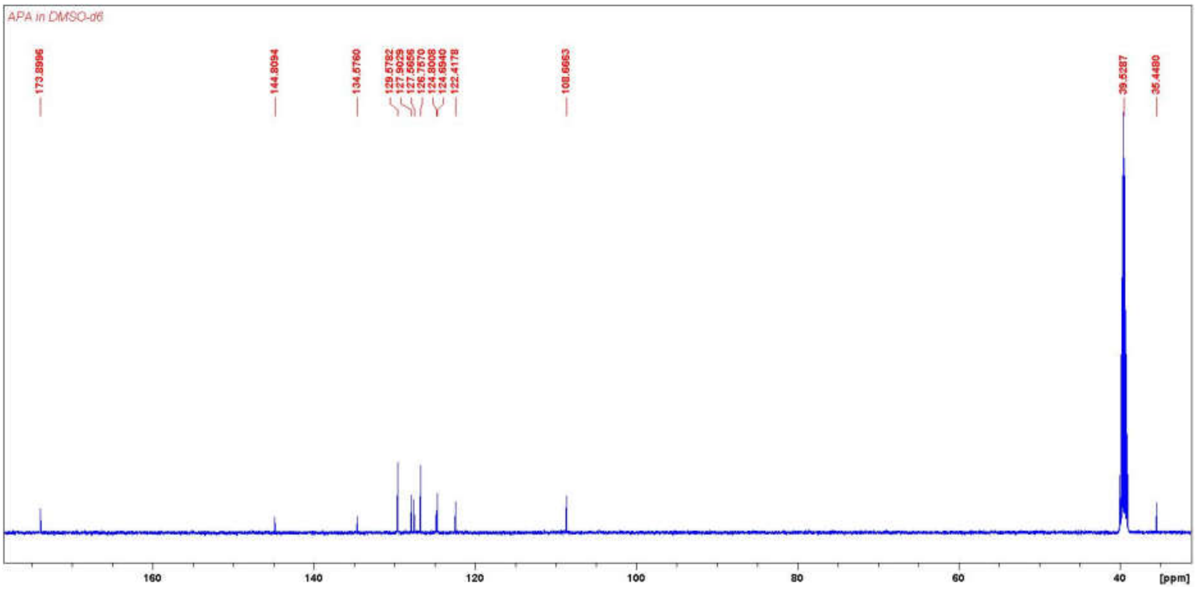

Figure S5. ^{13}C{^{1}H} NMR spectrum of APA in DMSO-d_6. δ (ppm): 173.9, 144.8, 134.6, 129.6, 127.9, 127.6, 126.8, 124.8, 124.7, 122.4, 108.7, 35.4. Resonance at 39.5 ppm corresponds to DMSO.

Upon the formation of APA, two new proton resonances attributable to H(8) (doublet, integral intensity of 2) appeared at 7.42 ppm in the corresponding ^{1}H NMR spectrum (Fig. S4). Surprisingly, the original NH resonance found at 10.32 ppm for DCF is missing in the case of the APA ^{1}H NMR spectrum, but this discrepancy was also observed earlier in the literature [59], and it might be caused by the extreme broadening of the signal in the coordinating solvent or by fast H for D exchange. On the other hand, when NMR spectra of APA are measured in $CDCl_3$, the NH fragment resonates as a very broad signal at 5.65 ppm (Figs. S6 and S7). In

general, the conversion of starting DCF to APA may be evidenced simply by the comparison of ^{1}H and $^{13}C\{^{1}H\}$ NMR spectra of the respective species since a totally different spectral pattern in the aromatic region is observed (Figures. S8 and S9).

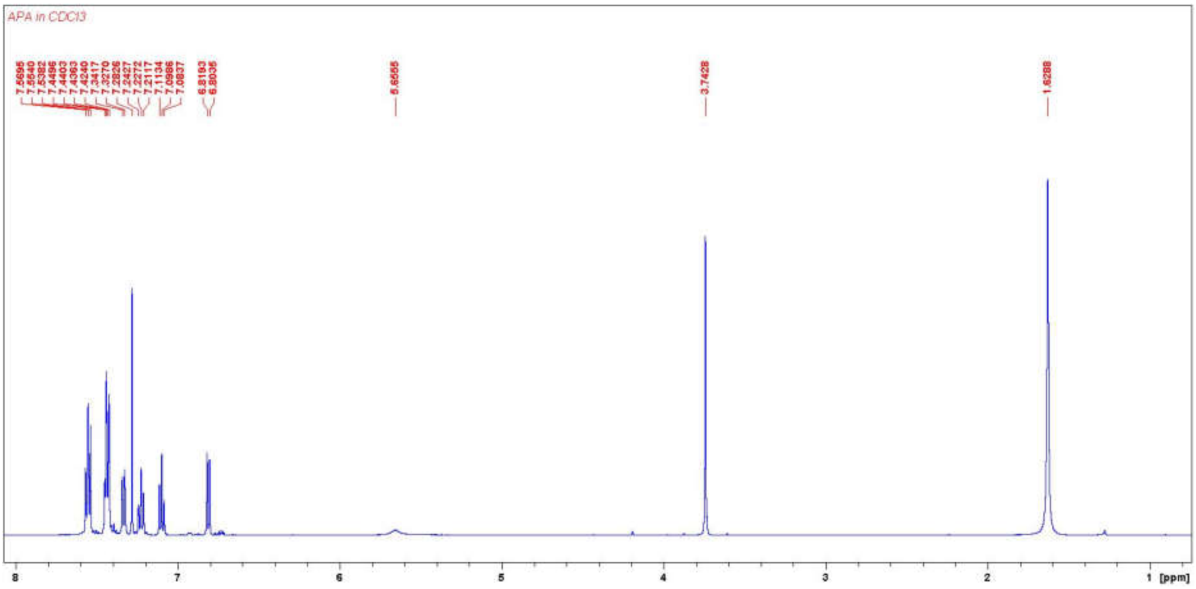

Figure S6. ^{1}H NMR spectrum of APA in $CDCl_3$. δ (ppm): 7.55 (m, 2H), 7.44 (m, 3H), 7.33 (d, 1H), 7.23 (t, 1H), 7.09 (t, 1H), 6.80 (d, 1H), 5.65 (broad, 1H), 3.74 (s, 2H). Resonances at 7.27 and 1.63 ppm correspond to residual $CHCl_3$ and water, respectively.

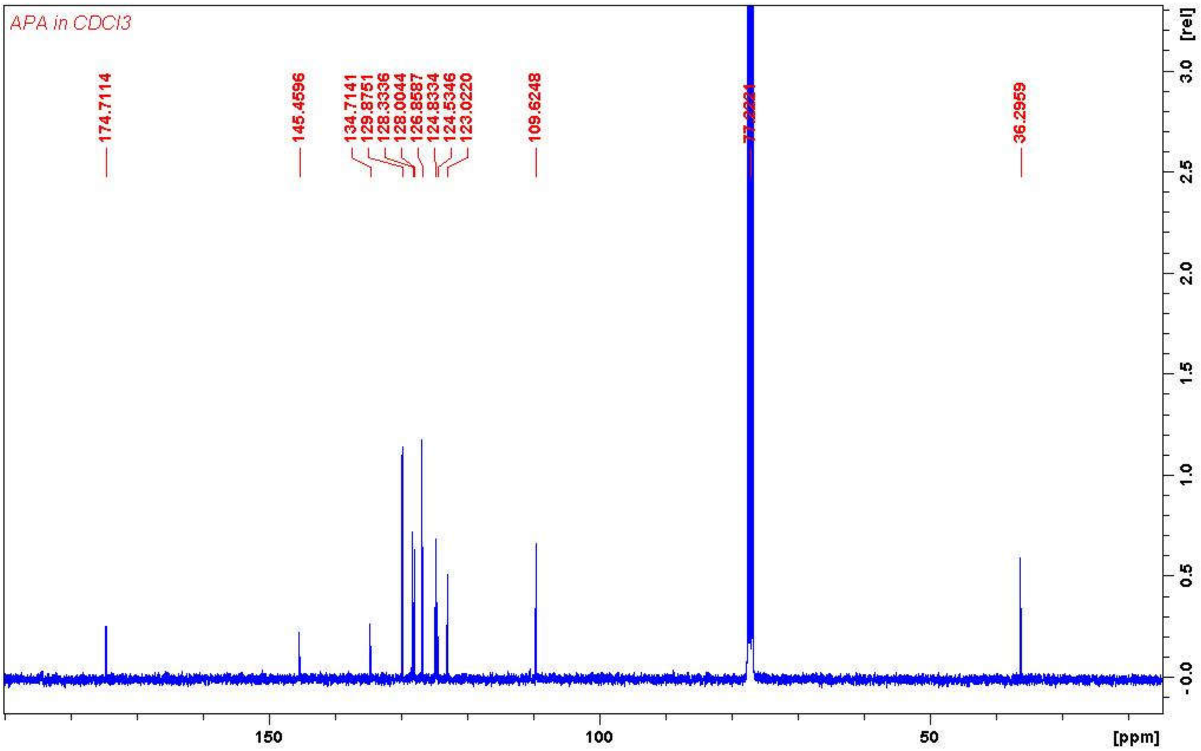

Figure S7. $^{13}C\{^{1}H\}$ NMR spectrum of APA in $CDCl_3$. δ (ppm): 174.7, 145.5, 134.7, 129.9, 128.3, 128.0, 126.8, 124.8, 124.5, 123.0, 109.6, 36.3. Resonance at 77.2 ppm corresponds to $CDCl_3$.

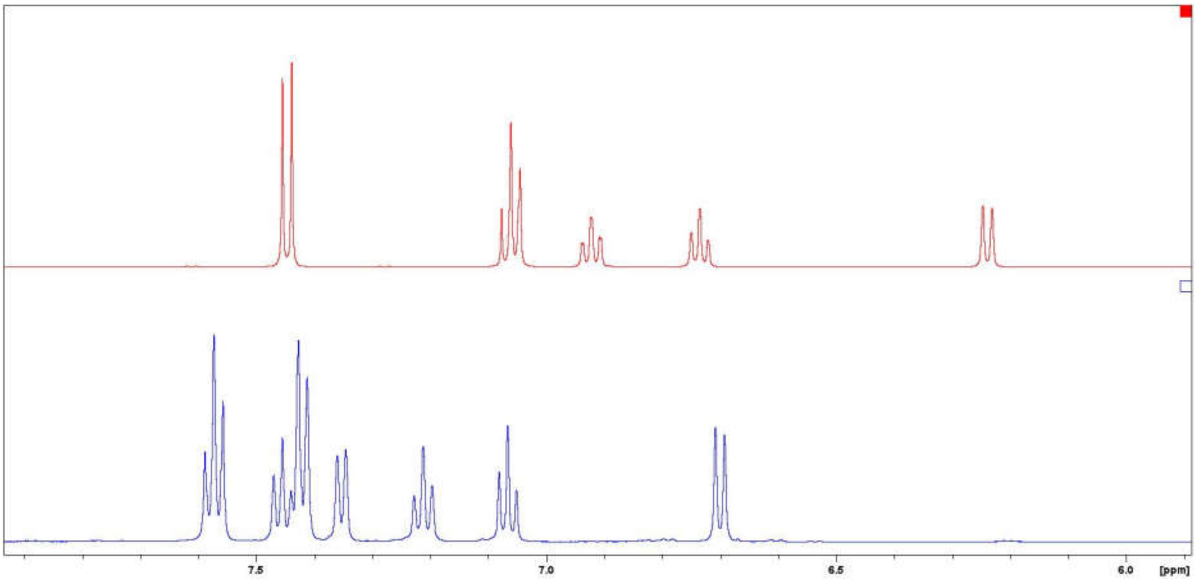

Figure S8. Comparison of aromatic regions of ^{1}H NMR spectra of starting DCF (red) and product of its hydrodechlorination, APA (blue).

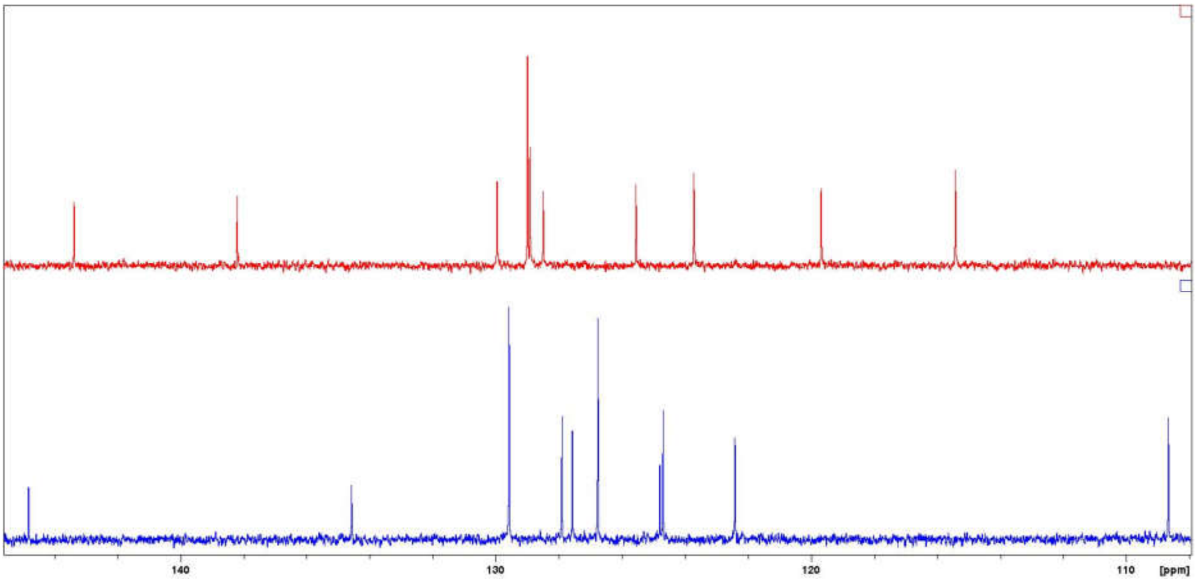

Figure S9. Comparison of aromatic regions of $^{13}C\{^{1}H\}$ NMR spectra of starting DCF (red) and product of its hydrodechlorination, APA (blue).

LC-MS/MS used for quantification of biocides:

The LC-MS/MS analyses of biocide samples were carried out with Nexera XR ultra-high-performance liquid chromatography (Shimadzu) and Sciex QTrap 4500 mass spectrometer (Amedis). The UHPLC was equipped with Cortecs T3 column (150 mm x 3.0 mm, particle diameter 2.7 µm, Waters) at a flow rate of 0.4 mL/min using 0.1% solution of formic acid in water (A) and acetonitrile (B) in following gradient: 0 min—20% B; 0.5 min—10% B; 8 min—70% B; 9 min—100% B; 12 min—100% B; 12.5 min—10% B; and 15 min—10% B. The pH of water samples delivered to the laboratory was adjusted to 3 using hydrochloric acid and filtered through a cellulose nitrate membrane filter (pore size 0.45 µm). The OASIS HLB 6cc cartridges (Waters, USA) were used for solid-phase extraction. Cartridges were conditioned with 3 mL of methanol and 3 mL of Milli-Q water. Five hundred milliliters of prepared water sample was loaded with the flow rate of about 50 mL min^{-1}. After sample application, cartridges were washed with 4 mL of 5% MeOH and subsequently eluted with 4 mL of MeOH. Eluate was evaporated to dryness under a gentle stream of nitrogen and redissolved in 1 mL of 50% MeOH. The analytes were detected in both negative ion mode (Hydrochlorothiazide, Diclofenac, Triclosan) and positive ion mode (Fluconazole, Chlorhexidine, Simazine, Ketoconazole, Cetirizine and Atrazine). The source block was maintained at 400 °C, and ion spray voltage was set at -4500 V for negative ion mode and +4500 for positive ion mode. A multiple reaction monitoring experiment (MRM) was set up using the characteristic transitions shown in Table S1. Structures of mentioned halogenated compounds are depicted in Table S2.

Table S1. Characteristic MRM transitions of targeted analytes.

Analytes	Q1		Q3	
	$[M+H]^+$	$[M+H]^-$	quantity	quality
Hydrochlorothiazide	-	295.8	204.9	77.8
Fluconazole	307.0	-	238.0	220.0
Chlorhexidine	253.1	-	169.9	177.0
Simazine	202.0	-	124.0	103.9
Ketoconazole	531.0	-	243.9	255.1
Cetirizine	389.1	-	200.9	166.0
Atrazine	216.0	-	103.9	131.9
Diclofenac	-	293.8	214.2	177.8
Triclosan	-	286.7	34.9	112.9

Table S2. Structures and formulas of biocides mentioned in Table S1

Name of biocide	Structure	Formula
Hydrochlorothiazide	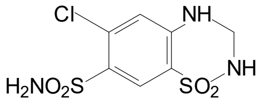	$C_7H_8ClN_3O_4S_2$
Fluconazole	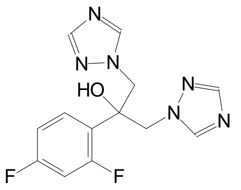	$C_{13}H_{12}F_2N_6O$
Chlorhexidine	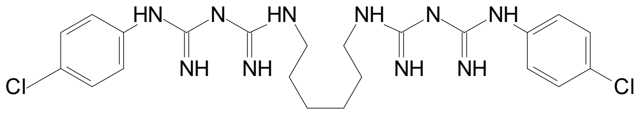	$C_{22}H_{30}Cl_2N_{10}$
Simazine	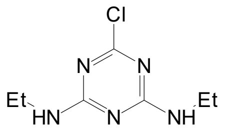	$C_7H_{12}ClN_5$
Ketoconazole	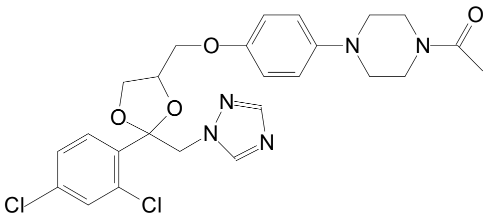	$C_{26}H_{28}Cl_2N_4O_4$
Cetirizine	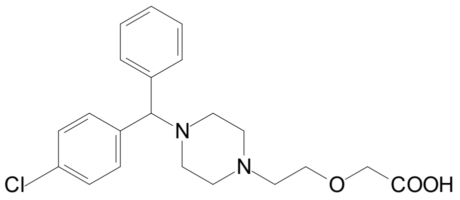	$C_{21}H_{25}ClN_2O_3$
Atrazine	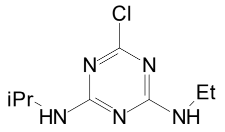	$C_8H_{14}ClN_5$
Diclofenac	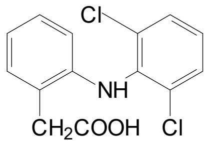	$C_{14}H_{11}Cl_2NO_2$
Triclosan	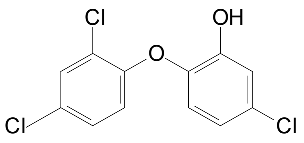	$C_{12}H_7Cl_3O_2$

Results:

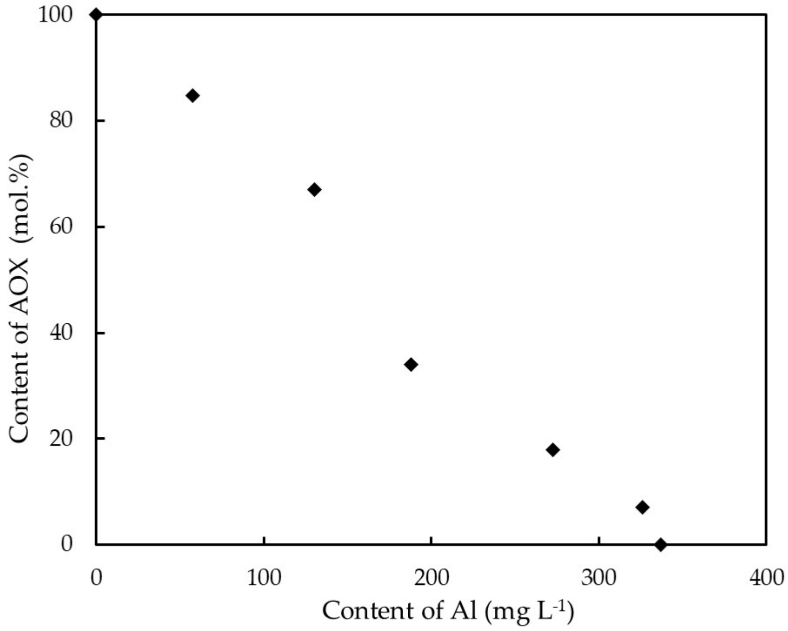

Figure S10. Dependence of decrease in AOX on quantity of dissolved Al; 6 mmol Al (in Al-Ni) + 30 mmol KOH per 0.4 mmol of DCF dissolved in 200 mL of water (see Table 1, run No.1).

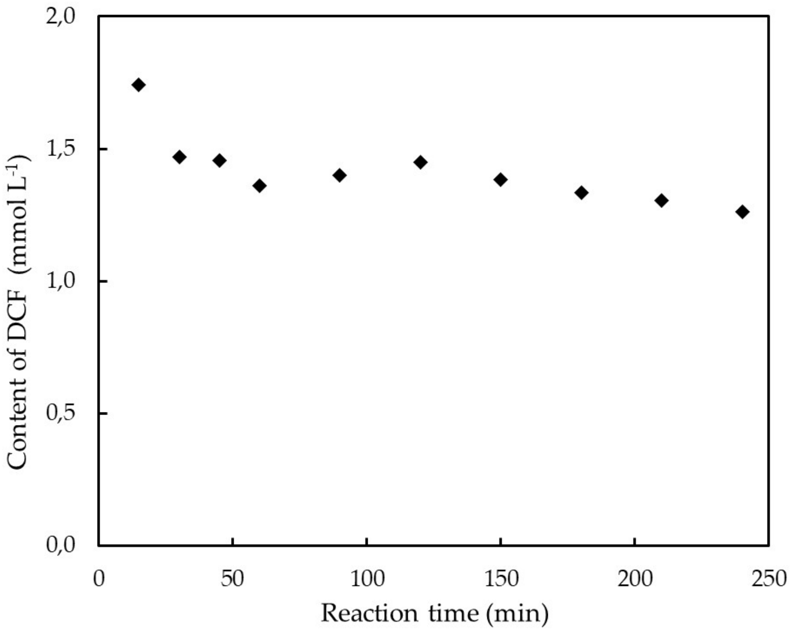

Figure S11. Adsorption of DCF onto the Al-Ni alloy in absence of KOH ([NaDCF] = 635 mg L^{-1} and [Al-Ni] = 1.65 g L^{-1}; 25 ^{o}C).

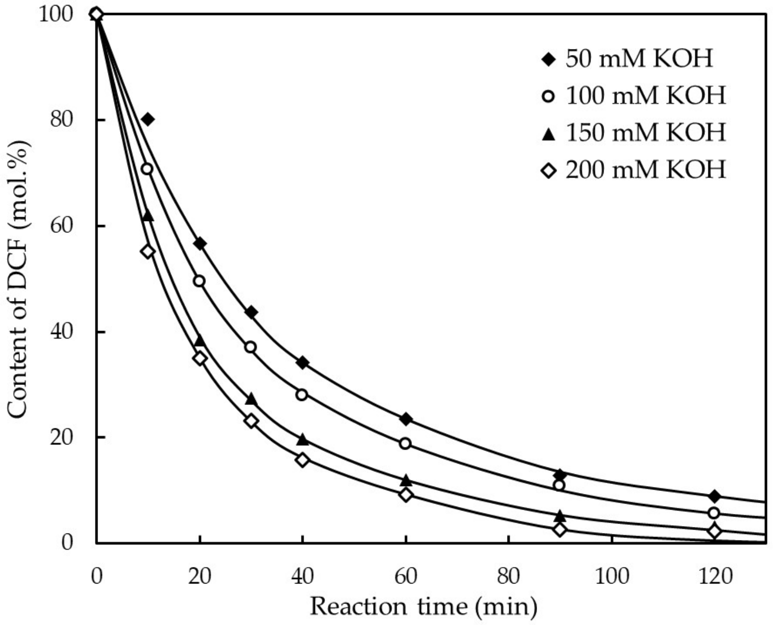

Figure S12. Comparison of the HDC rates of DCF using different amounts of KOH (0.4 mmol DCF + 4 mmol Al (in Al-Ni)).

X-ray measurements:

Experimental:

The diffraction patterns (Cu Kα, λ = 1.5418 Å) of powdered samples were measured using a D8 Advance diffractometer (Bruker AXS, Karlsruhe, Germany). The mass concentrations of the present phases were calculated by full pattern matching using EVA software [60]. For each selected pattern, EVA adjusts the scaling factor and width parameters by fitting, and the result is transformed into concentrations using the I/Icor factors from the PDF-4+ database [61].

Results:

The starting Raney Al-Ni alloy and its reaction products were studied by X-ray powder diffraction (see Fig. S12a-d). The crystalline phases present were determined by comparison with standards in the PDF-4+ database [8]. For multiphase samples, the mass content of the individual components was calculated. The starting Raney Al-Ni alloy contained 62 % Ni_2Al_3 (JCPDS No. 04-083-0990) and 38 % $NiAl_3$ (JCPDS No. 04-083-0990). The $NiAl_3$ diffraction lines disappeared completely, the Ni_2Al_3 content decreased, and broad diffraction lines of poorly crystalline phase $Ni_{0.879}Al_{0.121}$ (JCPDS No. 04-007-0414)) appeared in the samples of the alloy used in HDC of DCF (run No. 7) and eroded alloy after the slower HDC influenced by the addition of glucose (run No. 8). The content of $Ni_{0.879}Al_{0.121}$ (JCPDS No. 04-007-0414) phase was 92 % for the alloy used in run No. 7 and 62 % for the eroded alloy after the glucose-assisted HDC process (run No. 8). The sample obtained by reaction with $NaOH/NaBH_4$ (run No.14) contains either the cubic phase $Ni_{0.879}Al_{0.121}$ or elemental Ni (JCPDS No. 04-016-4592).

SEM

Figure S12 shows XRDs including approximate Al content of the fresh and used Al-Ni alloy particles applied in this study without and with the addition of glucose (Fig. S12a-c), Fig. S12d action of $NaBH_4$ in NaOH (used in excess) on used Al-Ni alloy (Run No. 15 in Table 1).

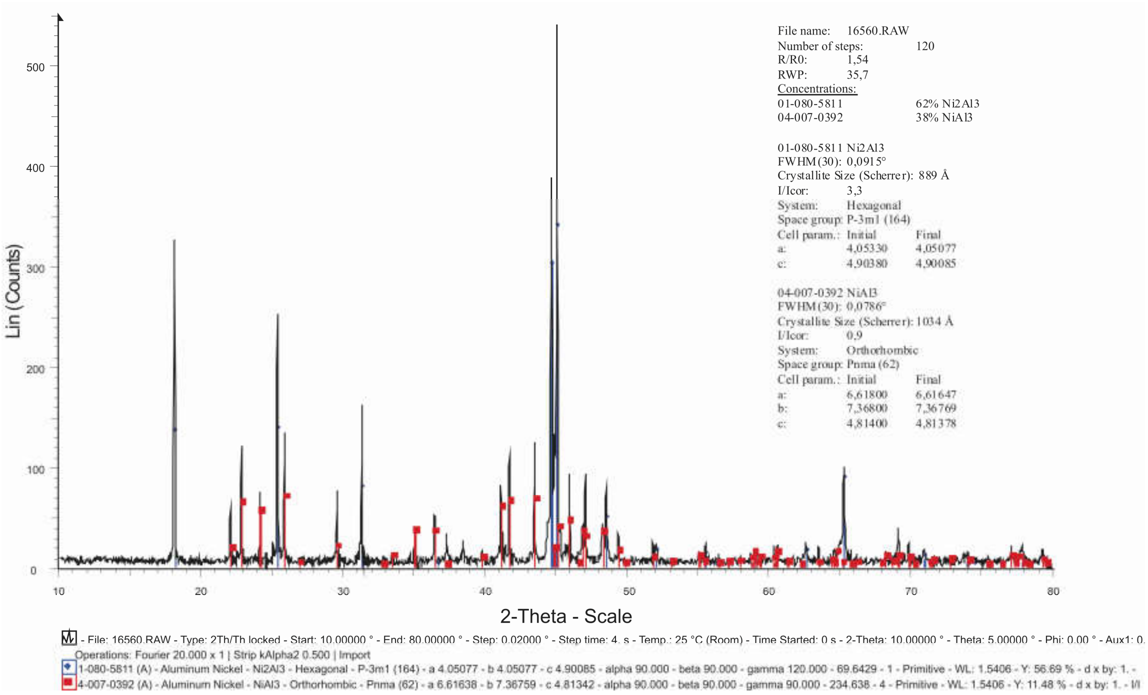

Figure 12a. XRD spectrum of starting commercial Raney Al-Ni alloy with composition ca. 62% Ni_2Al_3 + 38% $NiAl_3$**.**

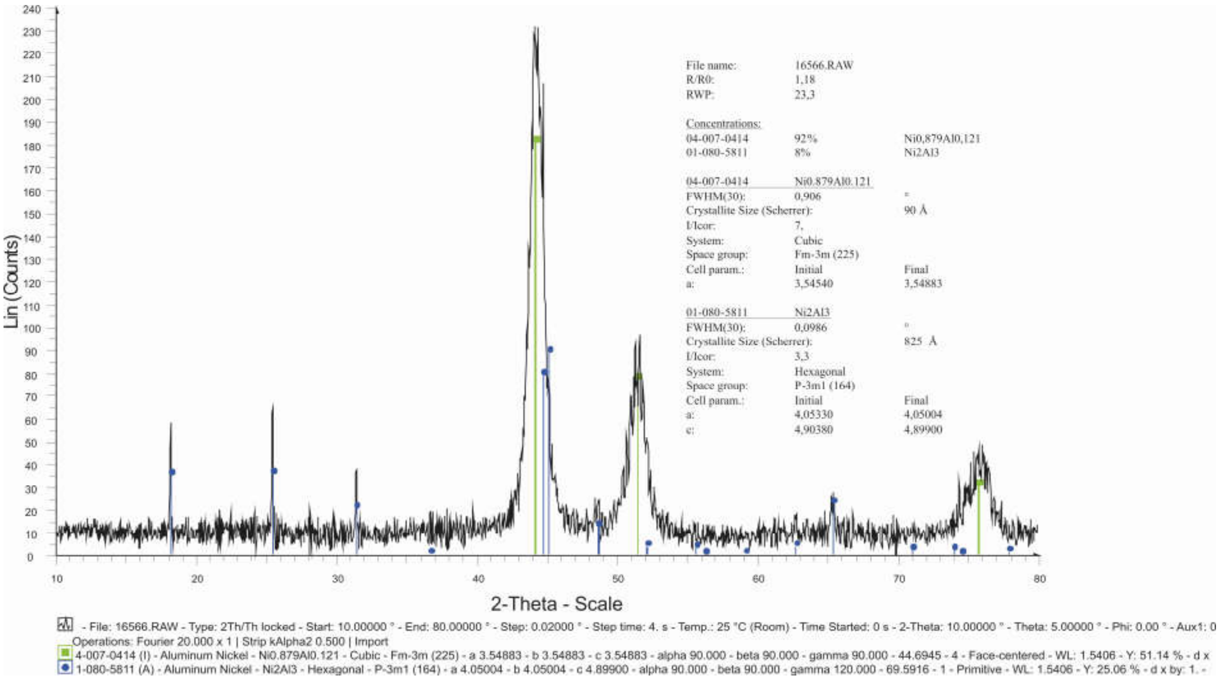

Figure 12b. XRD of eroded Al-Ni alloy after hydrodechlorination process (run No. 7 in Table 1) still contains some Al: 8% Ni_2Al_3 + 92% $Ni_{0.879}$ $Al_{0.121}$.

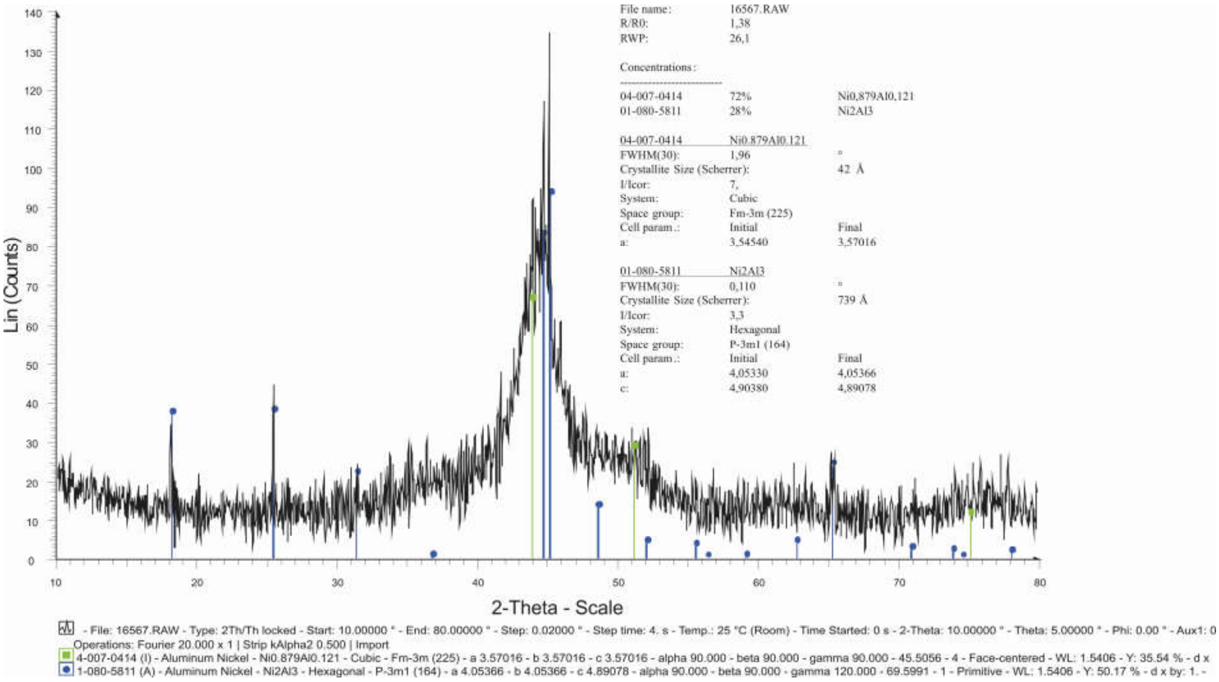

Figure 12c. XRD of used Al-Ni alloy in co-action of glucose, run No. 8 in Table 1: 72% $Ni_{0.879}Al_{0.121}$ + 28% Ni_2Al_3 (less Al is dissolved compared with run 7 in Table 1).

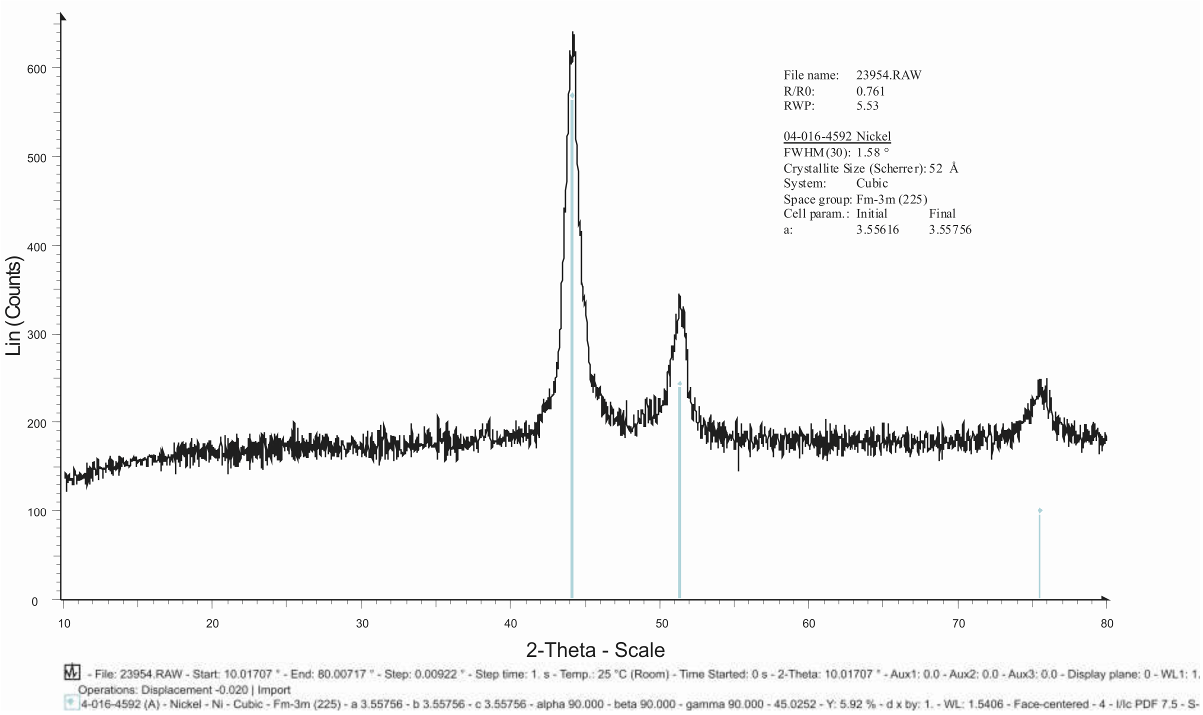

Figure 12d. XRD of Ni slurry (cubic Ni^0) obtained by action of $NaOH/NaBH_4$ (run No.15 in Table 1).

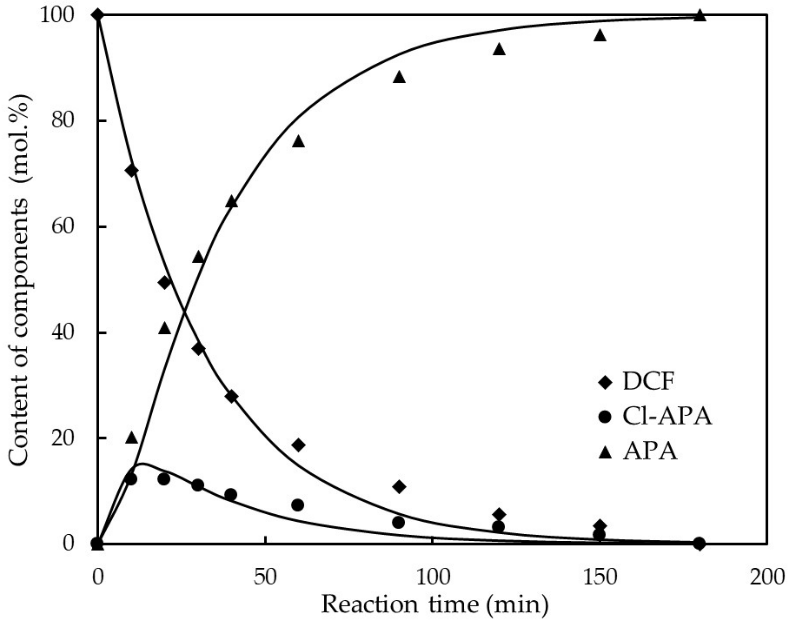

Figure S13. Results of experiment No. 7, mentioned in Table 1 (0.4 mmol DCF + 4 mmol Al (in Al-Ni) + 20 mmol KOH). Experimental (symbols) and model fit (solid lines).

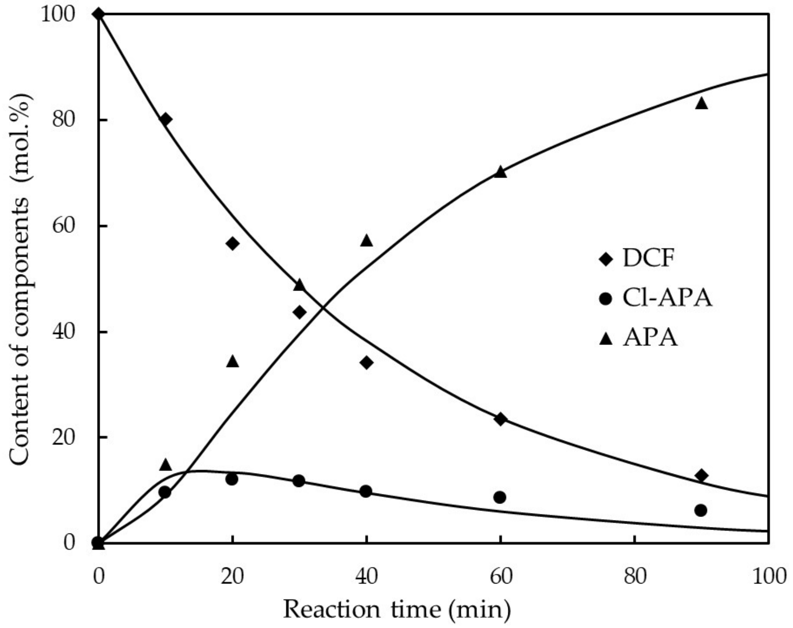

Figure S14. Results of experiment No. 10 in Table 1 (0.4 mmol DCF + 4 mmol Al (in Al-Ni) + 10 mmol KOH). Experimental (symbols) and model fit (solid lines).

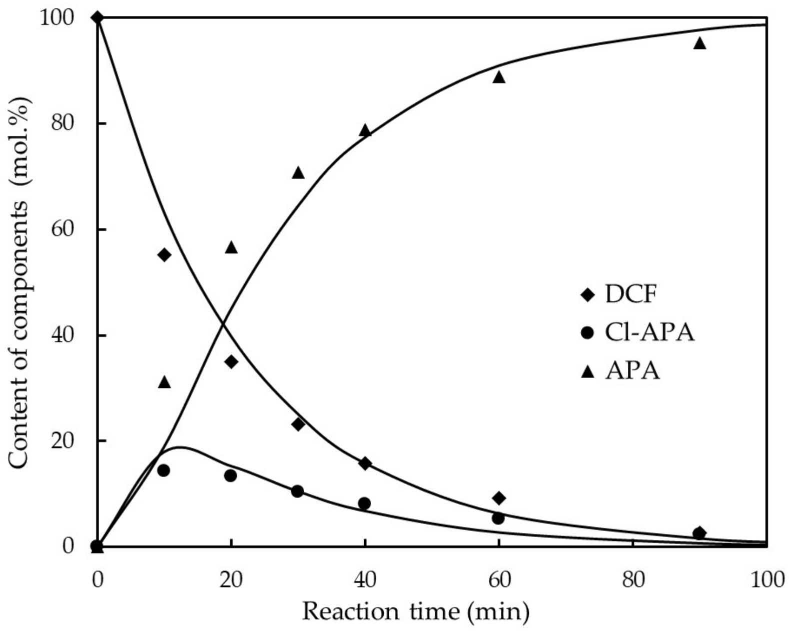

Figure S15. Results of experiment No. 5 in Table 1 (0.4 mmol DCF + 4 mmol Al (in Al-Ni) + 40 mmol KOH). Experimental (symbols) and model fit (solid lines).

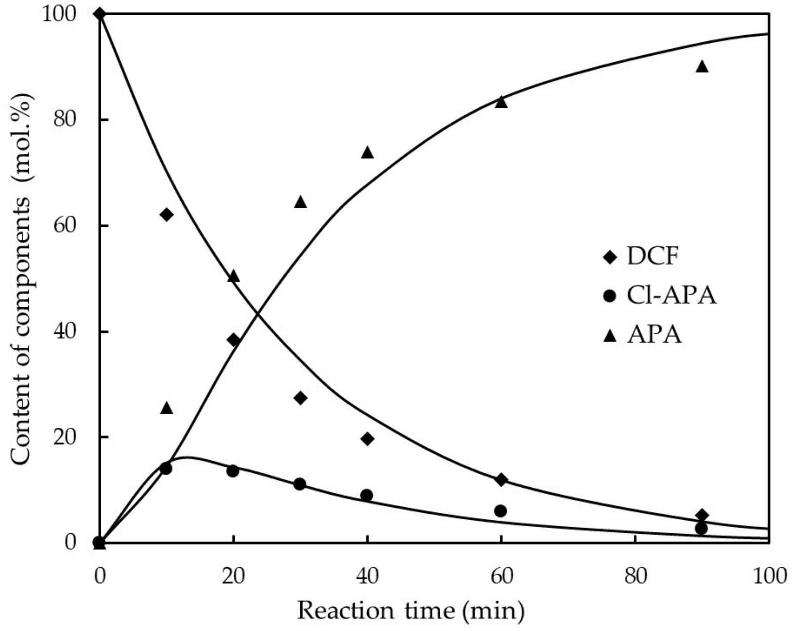

Figure S16. Results of experiment No. 6 in Table 1 (0.4 mmol DCF + 4 mmol Al (in Al-Ni) + 30 mmol KOH). Experimental (symbols) and model fit (solid lines).

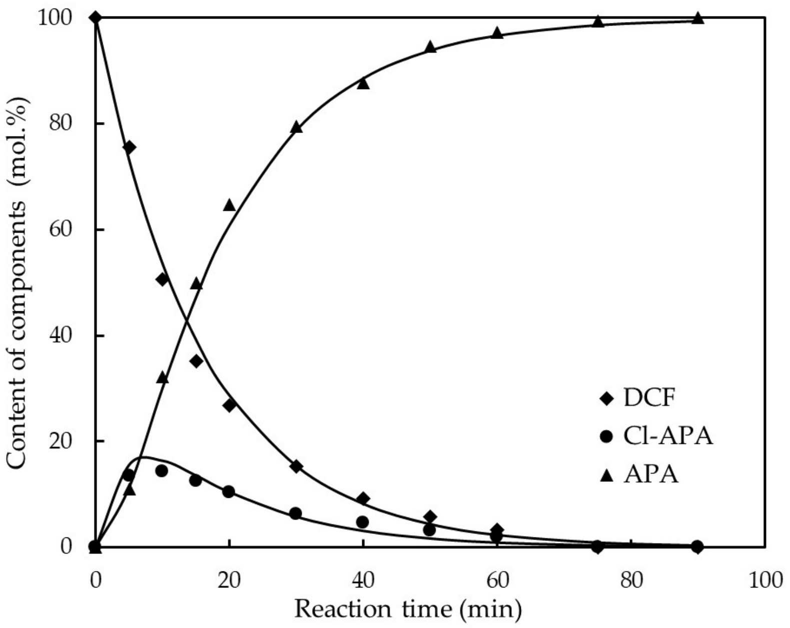

Figure S17. Results of experiment No. 4 in Table 1 (0.4 mmol DCF + 5 mmol Al (in Al-Ni) + 25 mmol KOH). Experimental (symbols) and model fit (solid lines).

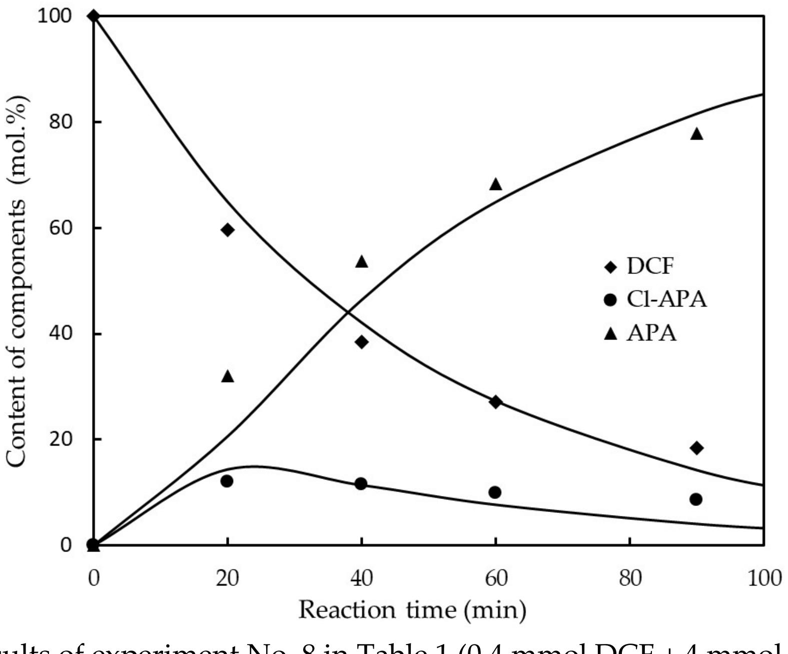

Figure S18. Results of experiment No. 8 in Table 1 (0.4 mmol DCF + 4 mmol Al (in Al-Ni) + 5 mmol glucose + 20 mmol KOH). Experimental (symbols) and model fit (solid lines).

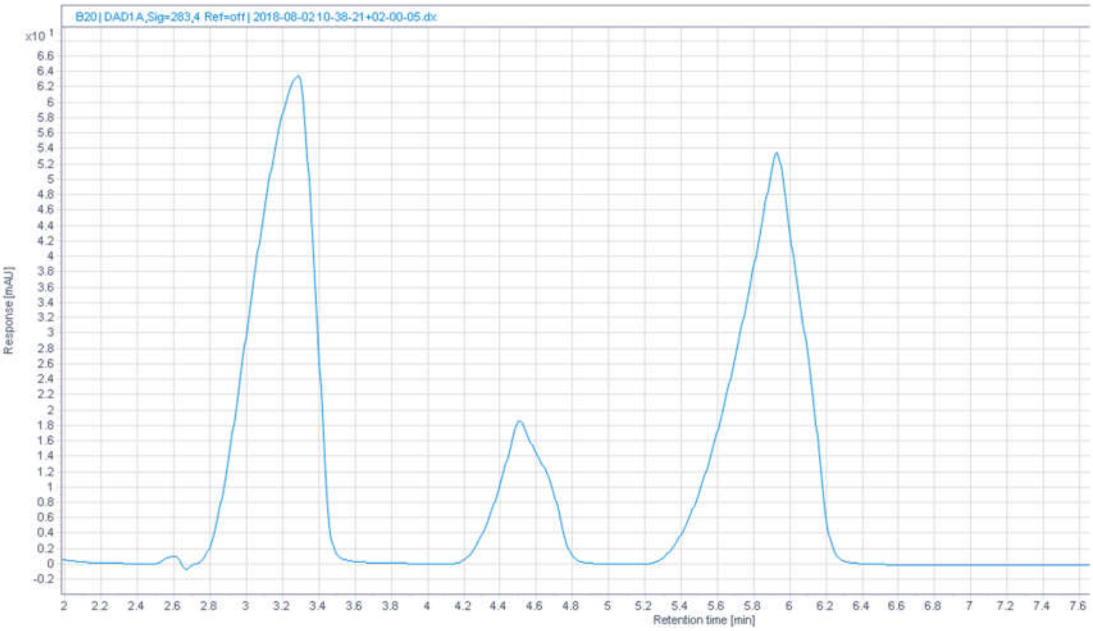

Figure S19. Example of chromatogram from HPLC, experiment No. 3 from Table 1, reaction time 20 min. First signal corresponds to APA (retention time 3.2 min.), second to Cl-APA (retention time 4.5 min.) and third to DCF (retention time 5.9 min.).
